# TiO_2_ nanoparticles assembled on kaolinites with different morphologies for efficient photocatalytic performance

**DOI:** 10.1038/s41598-018-29563-8

**Published:** 2018-08-03

**Authors:** Xiaoyu Li, Kang Peng, Huaxin Chen, Zhenjun Wang

**Affiliations:** 1 School of Materials Science and Engineering, Chang’an University, Xi’an, 710064 China; 2 State Key Laboratory for Mechanical Behavior of Materials, Xi’an Jiaotong University, Xi’an, 710049 China

## Abstract

Natural kaolinite clays with different dimensionalities (including kaolinite nanoflakes and nanorods) supported TiO_2_ nanoparticles were successfully prepared via a facile sol-gel method. Moreover, comparisons between FK/TiO_2_ and RK/TiO_2_ nanocomposites are conducted in terms of matrix morphology, surface property, energy band structure and interfacial interaction. The effects of kaolinite microstructure, morphology and dimensionality on the interfacial characteristics and photocatalytic properties of the nanocomposites were investigated in detail. The results showed that TiO_2_ nanoparticles are more easily attached on the kaolinite nanoflakes, and possess more uniform distribution and smaller particle size than that of kaolinite nanorods. In particular, the FK/TiO_2_ nanocatalysts exhibit higher photocatalytic activity for the degradation of tetracycline hydrochloride than that of RK/TiO_2_ and bare TiO_2_, which is attributed to the stronger surface adsorptivity, higher loading efficiency and smaller grain size. Additionally, FK/TiO_2_ composites show excellent stability, which is ascribed to the intimate interfacial contact between two-dimensional kaolinite nanoflakes and TiO_2_ nanoparticles. Overall, the enhanced catalytic performance for FK/TiO_2_ composites is the synergistic effect of two-dimensional morphology, better adsorption capability and more active photocatalysis TiO_2_ species.

## Introduction

Heterogeneous nanocomposites has attracted increasing attention because of the synergetic properties and potential applications as green methods to solve the energy and environmental problems^1^. In recent years, many technologies are proposed to tailor and promote the properties of nanocomposites, including element doping^2^, surface loading^3–6^, morphology controlling^7–11^, heterostructure constructing^12–16^, energy-band engineering^17^, and so on. Among them, loading functional nanoparticles on the surface of matrix materials is a promising alternative to control the nanoparticle size, and overcome inherent drawbacks of unsupported nanoparticles in terms of stability, agglomeration and reusability^18^.

As is well known, the performance of nanocomposites depends not only on the chemical composition, but also on microstructure, dimension, size and morphology and so on. At present, much interest has been focused on nanoparticles anchoring on a single support or the morphology-controlled synthesis of functional particles via different strategies, and further studied the comparative catalytic efficiency of the synthesized samples. Yang *et al*.^19^ synthesized a series of graphene-TiO_2_ nanocomposites with different TiO_2_ dimensionalities (including TiO_2_ nanoparticles, nanotubes and nanosheets) via sol-gel method, alkaline hydrothermal process and one-step solvothermal method, respectively. Meshram *et al*.^20^ reported synthesis of CuO nanostructures with different morphologies such as spherical, vesicular, nanosheet and platelet using chemical precipitation and hydrothermal methods. The photocatalytic activities of CuO nanostructures were evaluated by monitoring degradation of methylene blue, and the platelet-like CuO nanostructures were found to have the best catalytic activity. Thuy *et al*.^21^ prepared TiO_2_ particles with differnent morphologies via hydrothermal process, and investigated the morphological effect of TiO_2_ on photocatalytic degradation of organic dyes. However, none of these reports investigate any particular insights into the morphology of the support which has been reported to be as important as its internal structure. Like synthetic MCM-41, as common matrix materials^22^, have different morphologies, including mesoporous silica nanoparticles^23^, nanofibers^24^, nanotubes^25^, ordered hollow spheres^26^ or core-shell structured spheres^27^. It is significant to select an optimum morphology for a support to prepare a more efficient and reusable catalyst in a green process.

**Figure 1:**
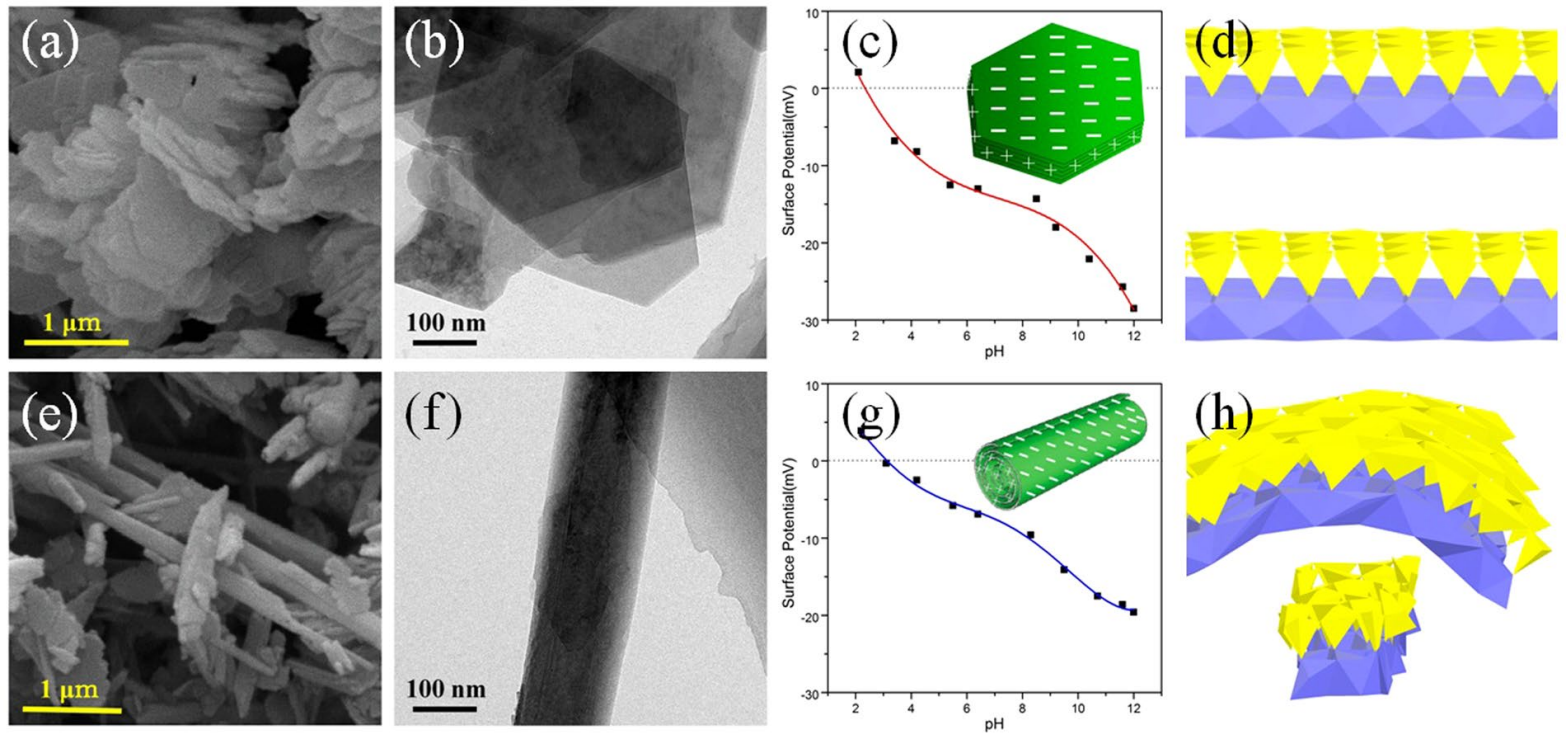
Morphology and schematic drawing of the samples. SEM, TEM images and Zeta potential (the insets are the schematic view of surface charge distributions) of (**a**–**c**) FK, (**e**–**g**) RK. Schematic drawing of the crystal structures of (**d**) FK and (**h**) RK as a combination of AlO_6_ octahedra with SiO_4_ tetrahedra.

Kaolinite [Al_2_Si_2_O_5_(OH)_4_] is a 1:1-type clay mineral composed of stacked layers of SiO_4_ tetrahedral sheets and AlO_2_(OH)_4_ octahedral sheets^28^ and naturally possesses diverse morphologies, including one-dimensional (nanotubes and nanorods) and two-dimensional morphology (nanoflakes)^29^. The schematic drawing of the crystal structures of kaolinite nanoflakes and kaolinite nanorods as a combination of AlO_6_ octahedra with SiO_4_ tetrahedra is shown in Fig. 1d,h. Meanwhile, kaolinite clays possess plentiful hydroxyl groups on the surface, which is beneficial to surface modification and make kaolinite become a suitable matrix for anchoring of TiO_2_ nanoparticles to enhance the photocatalytic activity. Zhang *et al*.^30^ successfully synthesized TiO_2_/kaolinite composites with mixed phase TiO_2_ (anatase and brookite) at low temperature. The crystal type of TiO_2_ nanoparticles could be controlled by the kaolinite matrix, and finally improve the catalytic performances. Hai *et al*.^31^ prepared acid-activated kaolinite via calcination and acid activation of coal-bearing kaolinite and then modified with TiO_2_ nanoparticles to improve its ability to adsorb and hence remove azo dyes. Kutlakova *et al*.^32^ prepared nanocomposite kaolinite/TiO_2_ using thermal hydrolysis of titanyl sulfate (TiOSO_4_) in the presence of kaolin, and calcined kaolinite/TiO_2_ at 600 °C. Thermal treatment was beneficial to the improvement of the photocatalytic activity of kaolinite/TiO_2_ composites. These studies are inclined to focus on the crystal structure changes of TiO_2_ on a substrate, but ignore the effect of matrix morphology on properties of TiO_2_. Therefore, preparing catalysts with kaolinites possessing the natural different morphologies is a very meaningful study to clarify the effect of clays dimensionality on the photocatalytic behavior and interfacial characteristics of kaolinite/TiO_2_ nanostructures.

In this work, based on the features of kaolin clays, unique layered structure, with similar chemical composition, but natural different morphologies (one-dimensional nanorods and two-dimensional nanoflakes), we have successfully assembled uniform TiO_2_ nanoparticles on the surface of kaolinite clays by a facile sol-gel method. The effects of kaolinite clays microstructure and dimensionality on the interfacial characteristics and catalytic properties of the composites were investigated in detail. TiO_2_ nanoparticles were efficiently deposited on the surface of kaolinites. The photocatalytic activity of this porous material was evaluated by tetracycline hydrochloride degradation. The clays/TiO_2_ simultaneously covered their excellent properties of TiO_2_ and kaolinites, exhibited high photocatalytic activity and adsorption property, and endowed this material with a bright perspective in degradation of antibiotics.

## Results

### Morphological and structural characteristics

**Figure 2:**
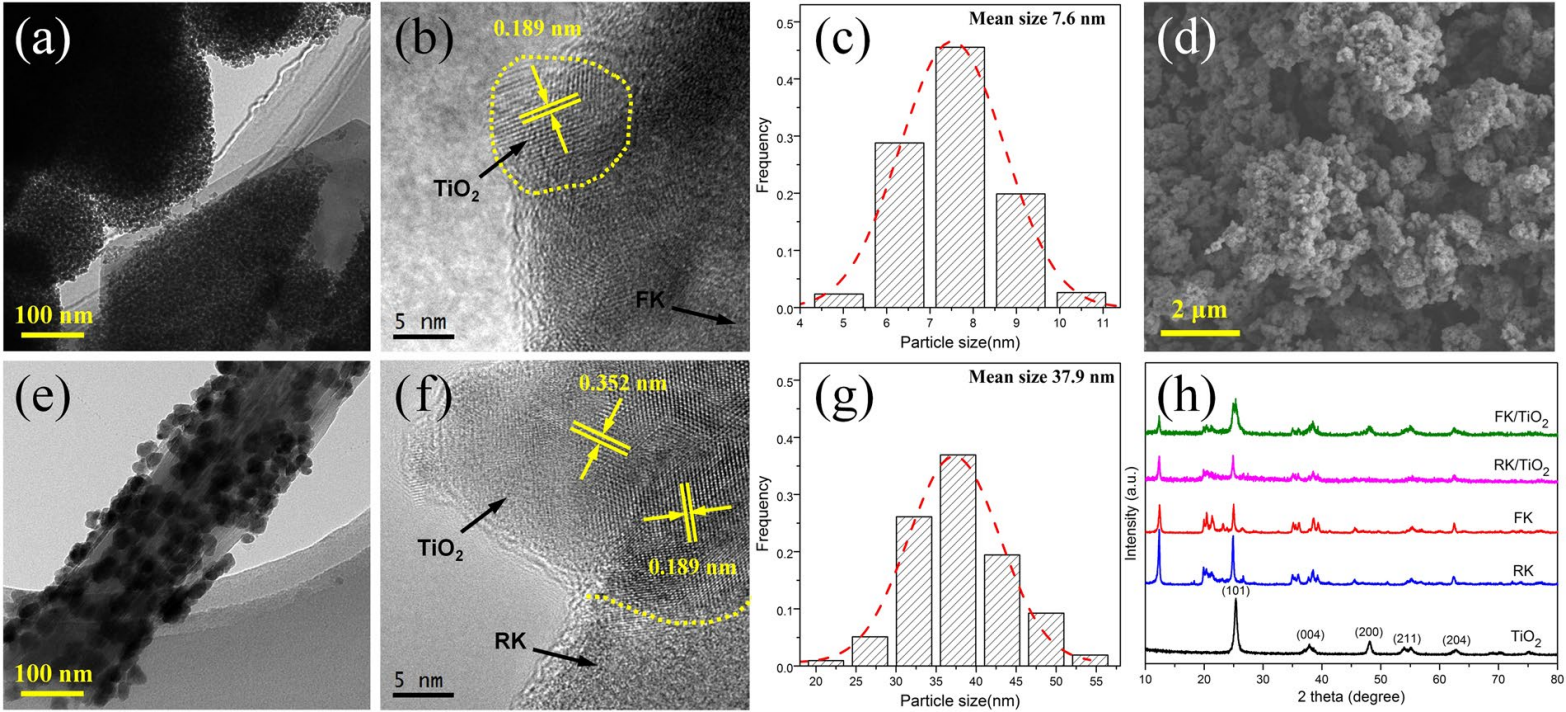
Morphology and crystallization of the samples. TEM, HRTEM images and the corresponding histograms of TiO_2_ clusters diameters of (**a**–**c**) FK/TiO_2_, (**e**–**g**) RK/TiO_2_. (**d**) SEM image of bare TiO_2_ aggregates, (**h**) XRD patterns of different samples.

The general morphologies of different samples were observed by electron microscope. Typical SEM and TEM analysis indicates that kaolinite clays used in the experiments naturally possess different morphologies, and the kaolinite particles all possess smooth surface without contamination (Fig. 1a,b,e,f). Kaolinite nanoflakes exhibit an irregular angular shape and the length of particles are in the range of microns (Fig. 1a,b). Nanorod-like kaolinites with smooth surface (Fig. 1e,f) are mostly 2~5 μm in length and 0.1~0.3 μm in diameter with a length to width ratio of about 20:1. It can be seen from Fig. 2d, bare TiO_2_ nanoparticles are obtained via the sol-gel method combined heat treatment. However, the dispersion of bare TiO_2_ nanoparticles is poor and exists obvious aggregation. In the presence of clays, the aggregation of TiO_2_ nanoparticles is markedly inhibited (Fig. 2a,e) and TiO_2_ nanoparticles are uniformly deposited on the surface of clays. After loading TiO_2_ nanoparticles, the clay supports show different loading results at the same experiment conditions. The TiO_2_ nanoparticles exhibit much more uniform and smaller for FK/TiO_2_ (Fig. 2a) and aggregation seriously for RK/TiO_2_ (Fig. 2e), which indicates that the morphology of supports have a seriously effect on the particles size. The HRTEM (Fig. 2b,f) results indicate that TiO_2_ nanoparticles have high crystallinity with well-defined lattice fringes of 0.189 nm corresponding to (200) plane, and 0.352 nm corresponding to (101) plane, in accordance with XRD results (Fig. 2h). Moreover, obvious interfaces are observed between the TiO_2_ and clays. The intimate interaction enables the electron to more easily transfer from TiO_2_ nanoparticles to kaolinite nanoflakes during the photoexcitation process. Meanwhile, the mean size of TiO_2_ nanoparticles for FK/TiO_2_ and RK/TiO_2_ measured on TEM images (Fig. 2a,e) are 7.6 and 37.9 nm, respectively, as shown in Fig. 2c,g. It is clear that the grain size of TiO_2_ nanoparticles is strongly depended on the morphology of the supports. Meanwhile, the TiO_2_ nanoparticles on kaolinite clays are much smaller than that of bare TiO_2_ (Fig. 2d), indicating the dispersion effect on TiO_2_ clusters on the surface of kaolinite clays. The measured surface zeta potential curves and the surface charge distributions sketches of pristine kaolinites with different morphologies are showed in Fig. 1c,g. It is clear that kaolinites with different morphologies carry a net negative charge, which allows for its good dispersibility in water and provided a strong electrostatic adsorption force to the positively charged molecules on the surfaces. But they possess the different surface charge distributions, thus leading to the different surface natures, which allows the adjustable grain size of TiO_2_ nanoparticles and further affects the photocatalytic performances.

As a result, the kaolinite clays with different morphologies can serve as supporting materials for *in situ* growth of TiO_2_ nanoparticles. The TiO_2_ nanocrystals attached on kaolinite nanoflakes have better dispersion and much smaller grain size than that of RK/TiO_2_ and bare TiO_2_ samples, resulting from the intimate interaction and good interfacial contact. Therefore, the TiO_2_ particles size can be controlled by clays and form interaction with clays surface, which would lead to exposing more catalytic reaction sites, improving photoelectrons transiting and enhancing the photocatalytic activity.

Figure 2h shows the XRD patterns of as-synthesized samples to study the crystal structure and crystalline phase of TiO_2_ in the nanocomposites. It can be clearly seen that kaolinite clays possessed the natural different morphologies have the similar crystal structure and their XRD patterns are in good agreement with the standard PDF card of JCPDS 14-0164 for kaolinite-1A^33^. The XRD pattern of bare TiO_2_ shows the highly crystalline anatase phase TiO_2_ (JCPDS No. 21–1272)^34^. The diffraction peaks of FK/TiO_2_ and RK/TiO_2_ composites are in good agreement with the anatase phase TiO_2_ and kaolinite, showing that the structure of kaolinite is maintained during the introduction of TiO_2_ nanoparticles. Meanwhile, the diffraction intensities of kaolintes in FK/TiO_2_ and RK/TiO_2_ composites are decreased, indicating the successful loading of TiO_2_ on the surface of kaolinites. The average grain size of TiO_2_ nanoparticles for FK/TiO_2_ and RK/TiO_2_ estimated by the Scherrer’s formula are 9.1 and 32.4 nm, respectively, which is consistent with the results based on TEM images. Moreover, the grain size of bare TiO_2_ is 16.8 nm. By comparison to the standard JCPDS diffraction patterns of anatase phase TiO_2_, it can be seen that the diffraction peaks of TiO_2_ on FK/TiO_2_ are much more obvious than that of RK/TiO_2_, which indicates that FK has relatively higher loading efficiency for TiO_2_ nanoparticles at the same experiment conditions. In order to further confirm the results, elemental analysis (Table 1) was employed in the experiment. It is shown that the relative content of titanium in FK/TiO_2_ is higher than that of FK/TiO_2_, which is consistent with the XRD results.

**Table 1:** The chemical composition of different samples (mass %).

Samples	SiO_2_	Al_2_O_3_	Fe_2_O_3_	CaO	MgO	TiO_2_	K_2_O	Na_2_O	Ig.loss
FK	46.90	38.14	0.15	0.01	0.11	0.14	0.30	0.19	13.98
RK	46.78	38.51	0.96	0.04	0.03	0.05	0.19	0.02	13.82
FK/TiO_2_	23.75	19.14	48.21	0.11	0.01	0.06	0.21	0.12	8.39
RK/TiO_2_	29.51	20.32	42.16	0.53	0.02	0.01	0.11	0.01	7.33

**Figure 3:**
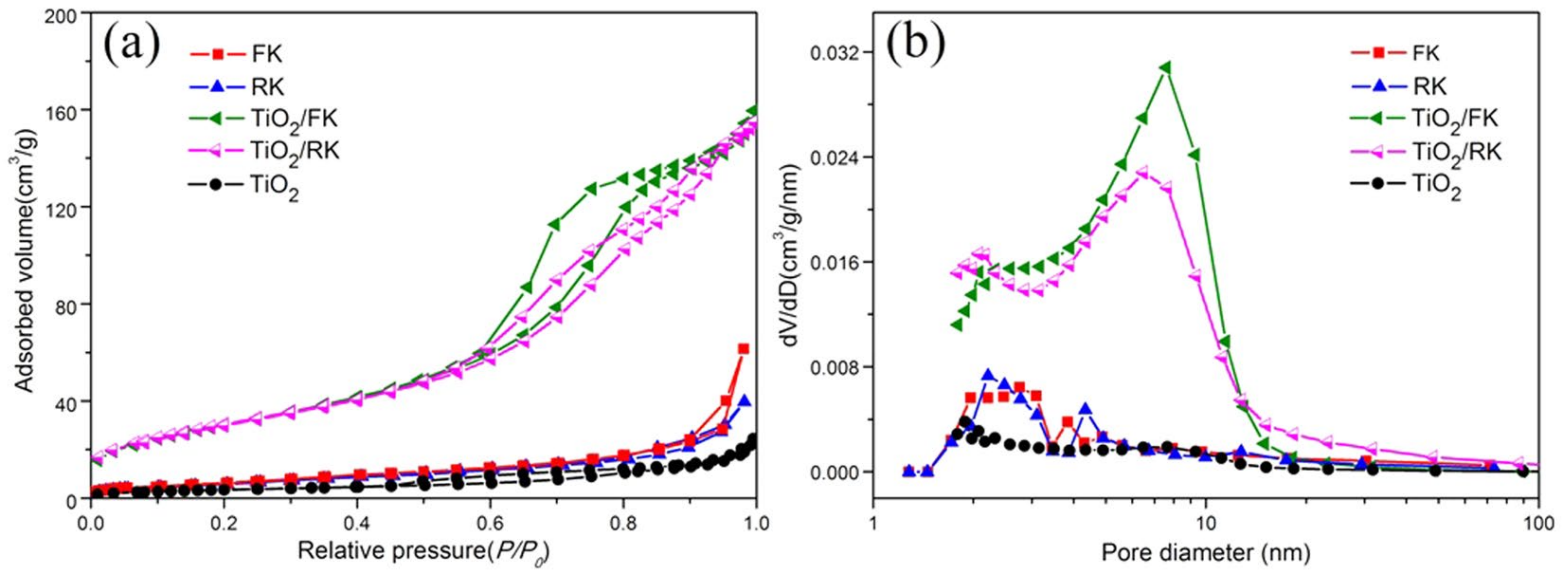
Textural characteristics of the samples. (**a**) Nitrogen adsorption-desorption isotherms, (**b**) corresponding BJH pore size distribution of different samples.

The nitrogen adsorption-desorption isotherms of different samples and BJH pore size distribution are shown in Fig. 3, and the textural parameters calculated from the corresponding isotherms are summarized in Table 2. As shown in Fig. 3a, the adsorption-desorption isotherms of bare TiO_2_ almost have no hysteresis loop, and the BET specific surface area is only 13 m^2^/g due to the seriously aggregation, while the adsorption-desorption isotherms of clays/TiO_2_ exhibit typical characteristic of type-IV with distinct type *H3* hysteresis loop, indicating the formation of mesoporous structures^35^. However, the adsorption-desorption isotherms of clays almost have no hysteresis loop, and the specific surface area are only about 27 m^2^/g, indicating clays nanopartiles can easily aggregate and result in the performance decline. After supporting TiO_2_ nanoparticles on the surface of clays, the BET specific surface area (*S*_BET_) and pore volume (*V*_pores_) of composites are increased, as summarized in Table 2. It shows that the mesopore structure can be formed after loading TiO_2_ nanoparticles by revealing increased surface area from 27 to 114 m^2^/g for FK/TiO_2_, and from 26 to 109 m^2^/g for RK/TiO_2_. The pore volume of FK/TiO_2_ and RK/TiO_2_ are 0.25 and 0.35 cm^3^/g, respectively. Obviously, FK/TiO_2_ has relatively higher specific surface area and lower pore volume, compared with RK/TiO_2_, which might be because the smaller size and uniform distribution of TiO_2_ nanoparticles attached on kaolinite nanoflakes. These results are further confirmed by the observation from their pore size distribution calculated by BJH method (Fig. 3b). These characteristics seem to be responsible for enhanced catalytic activity and stability on the clays/TiO_2_ in comparison with bare TiO_2_. These properties are also attributed to the formation of small particles through oxide-support interaction to provide more active sites for photocatalysts.

**Table 2:** The textural characteristics of all samples.

Material	*S*_BET_ (m^2^/g)	*V*_pores_ (cm^3^/g)	*d*_pores_ (nm)	Band gap energy (eV)
FK	27	0.09	11.8	—
RK	26	0.06	9.2	—
FK/TiO_2_	114	0.25	8.7	3.21
RK/TiO_2_	109	0.35	12.5	3.19
TiO_2_	13	0.04	11.4	3.20

Notes: *S*_BET_ = BET specific surface area, *V*_pores_ = total pore volume, *d*_pores_ = average pore diameter.

### Interfacial characteristics

The surface properties of TiO_2_ species play an important role in aqueous phase photocatalytic reaction, and a large surface area of FK/TiO_2_ is responsible for the efficient catalytic activity. In addition, the interactions between TiO_2_ and clays are also important to suppress the aggregation of TiO_2_ nanoparticles during the reaction, and the oxide-support interactions are measured by FTIR and XPS, which can provide additional information on the structure of clays/TiO_2_ nanocomposites.

**Figure 4:**
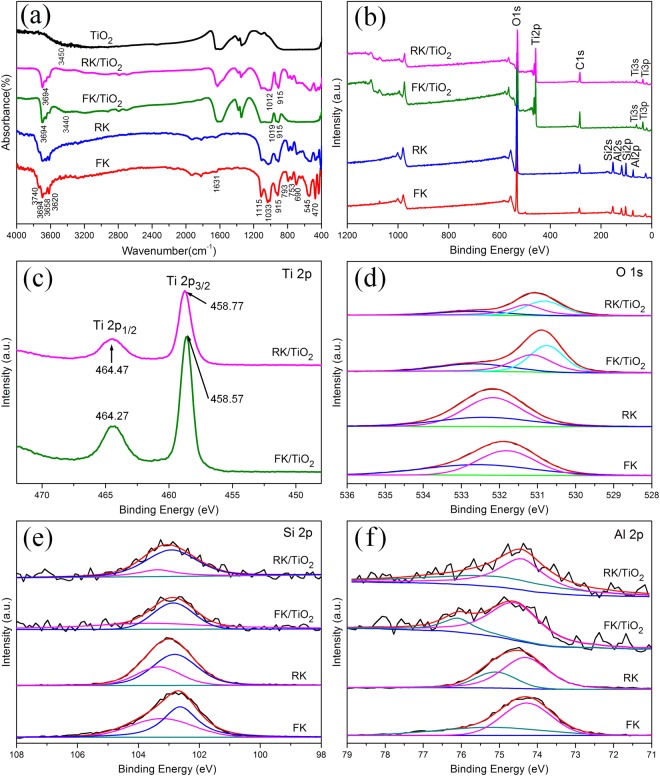
Interfacial characteristics of the samples. (**a**) FTIR spectra and (**b**) XPS survey spectra of different samples. (**c**) Ti 1 s, (**d**) O 1 s, (**e**) Si 2p and (**f**) Al 2p fitted XPS spectra of different samples.

The FTIR spectra of the samples are shown in Fig. 4a to analyze the vibrational bands and the interface interaction. For FK and RK, the peak at 1033 cm^−1^ corresponds to the stretching vibration of the skeleton Si-O network (Si-O-Si and O-Si-O)^36^. The broad band between 1631 cm^−1^ is assigned to adsorbed water. The other bands at 3658 and 1116 cm^−1^ are assigned to inner surface hydroxyl out-of-phase stretching vibration and apical Si-O stretching vibration, respectively. The absorption bands at 3694, 3620, and 915 cm^−1^ are ascribed to an inner-surface hydroxyl (Al-OH) stretching vibration, which are rarely influenced by intercalation^29^. The peak at 3740 cm^−1^ is assigned to the outer-surface hydroxyl (Si-OH) stretching vibrations. The bands at 793, 753 and 690 cm^−1^ are due to the translational vibration of O-Al-OH. Meanwhile, the bands at 545 and 470 cm^−1^ are attributed to the vibration of Si-O-Al. There is little change in the band positions of RK compared with FK, only the overall intensity are slightly reduced. FK/TiO_2_ and RK/TiO_2_ show the characteristic bands of kaolinite at 3694, 1116 and 915 cm^−1^ and TiO_2_ at between 1600 and 1200 cm^−1^. The absorption band at 3740, 3658 and 3620 cm^−1^ ascribed to surface hydroxyl groups vibration are disappeared, indicating the structural dehydroxylation of the kaolinite, which might be caused by immobilization of TiO_2_ nanoparticles on the surface of kaolinite or the bonding on Ti atoms on these sites, leading to a reduction of hydroxyl groups. The Si-O stretching band at about 1033 cm^−1^, which shifts to 1019 and 1012 cm^−1^ for FK/TiO_2_ and RK/TiO_2_, respectively. The shifting of the skeleton Si-O network stretching band indicates the formation of hydrogen bonding between the outer surface of the kaolinites (tetrahedral sheet) and TiO_2_. Moreover, the new broad band around 3440 cm^−1^ is assigned to Ti-O and its intensity for FK/TiO_2_ is higher than that of RK/TiO_2_. Furthermore, the bands at 793, 753, 690, 545 and 470 cm^−1^ are disappeared for FK/TiO_2_, while still remained for RK/TiO_2_. These results provide evidences for the existence of interaction between kaolinite and TiO_2_ and confirm that the FK can be coated by TiO_2_ nanoparticles much more effectively and the binding force is much stronger than that of RK, which is consistent with the TEM (Fig. 2) and XRF results (Table 1).

The interactions between TiO_2_ and kaolinite clays in the composites are further investigated by using XPS spectra. Figure 4b shows the XPS survey spectra of FK, RK, FK/TiO_2_ and RK/TiO_2_ in the range 0–1200 eV. The XPS survey spectra of FK/TiO_2_ and RK/TiO_2_ clearly indentify the signals of the Al, Si, Ti and O elements. The weak signals of Si and Al elements for clays/TiO_2_ indicate that the kaolinites surface are coated with a layer of dense nano-sized TiO_2_ particles.

The high-resolution XPS spectra of Ti 2p, O 1 s, Si 2p and Al 2p for samples are exhibited as Fig. 4c–f, respectively. As shown in the high-resolution spectra of Ti 2p electrons (Fig. 4c), two bands located at 458.77 and 464.47 eV for RK/TiO_2_, and 458.57 and 464.27 eV for FK/TiO_2_ can be assigned to Ti 2p_3/2_ and Ti 2p_1/2_ spinorbital splitting photoelectrons in the Ti^4+^ chemical state, respectively^37^. Meanwhile, Ti^4+^ combines with kaolinites and forms Si-O-Ti bond^37^. This interaction of chemical bond can immobilize TiO_2_ to prevent it from movement and agglomeration. The slight shifts for FK/TiO_2_ and RK/TiO_2_ can be due to a change in the chemical state or coordination environment of Ti 2p, that is, the interaction between kaolinites with different morphologies and TiO_2_ nanoparticles. Moreover, the larger peak area of Ti 2p for FK/TiO_2_ indicates that TiO_2_ nanoparticles are more easily loaded onto the surface of the flake-like support under the same condition.

The high resolution O 1 s spectra of kaolintes can be deconvoluted into two fitted peaks (Fig. 4d). The peak at around 532.6 eV can be assigned to lattice oxygen in the kaolintes, and the other peak at about 531.7 eV is derived from the hydroxyl group^38^. For clays/TiO_2_, the peaks at 532.6 eV and 530.6 eV are assigned to oxygen from Si-O-Si and Ti-O-Si, and the peak at 531.2 eV is assigned to oxygen from hydroxyl group. The result is in good consistent with that of Ti 2p (Fig. 4c) and confirms the existence of Ti-O-Si and surface hydroxyl. Moreover, the shifts of surface hydroxyl groups and the new formed Ti-O-Si demonstrate the integration between TiO_2_ and kaolintes and intense interaction between the two components. Moreover, the larger peak area of Ti-O-Si for FK/TiO_2_ further indicates FK/TiO_2_ has relatively higher loading efficiencyof TiO_2_ nanoparticles than that of RK/TiO_2_.

Figure 4e,f shows the Si 2p and Al 2p spectra of different samples, and the peak positions for kaolintes are observed at 103.3 eV (Si-OH), 102.7 eV (Si-O), 75.1 eV (Al-OH) and 74.3 eV (Al-O), respectively^39^. After supporting TiO_2_ nanoparticles on the surface of clays, the reduction of hydroxyl groups and slightly shifts for clays/TiO_2_ nanocomposites indicate the interaction between the two components.

### Optical spectroscopic study

**Figure 5:**
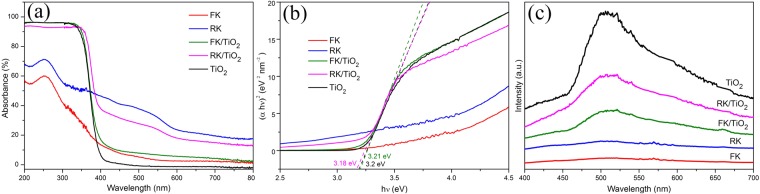
Optical spectroscopic study of the samples. (**a**) UV-vis diffuse reflectance spectra of different samples, (**b**) the corresponding plots of (*αhv*)^2^ vs. photon energy (*hv*). (**c**) The photoluminescence spectra of different samples.

The UV-vis diffuse reflectance spectra are used to determine the optical properties of the synthesized samples. As shown in Fig. 5a, the FK and RK show clear absorption in the UV region, and a visible light absorption around 500 nm for RK can be observed. It is obvious that bare TiO_2_ nanoparticles have no or relatively weak visible light absorption, while clays/TiO_2_ nanocomposites exhibit enhanced light absorption capacity in the UV-vis region, indicating that the weak absorption in the visible light region can be attributed to the kaolinites. In comparison to kaolintes, the absorptions attributed to crystalline TiO_2_ around 400 nm are present, which further confirms that the crystalline TiO_2_ nanoparticles are successfully attached on the surface of kaolinites in clays/TiO_2_ nanocomposites. Meanwhile, the positions of adsorption onsets of clays/TiO_2_ nanocomposites exhibit a significant shift compared to bare TiO_2_ nanoparticles, which is due to the quantum size effect and dispersion effect of kaolintes. The shift of adsorption edge for FK/TiO_2_ is stronger than RK/TiO_2_. It might be due to the smaller size of TiO_2_ in FK/TiO_2_, which is well consistent with the TEM results (Fig. 2).

The bandgap energy of clays/TiO_2_ samples can be confirmed roughly according to the plot of (*αhv*)^2^ versus energy (*hv*) of absorbed light (Fig. 5b), which is obtained on the basis of the Kubelka-Munk function (F(*R∞*) = (1 − *R*)^2^/(2 *R*)), where *α*, *h*, *v* and *R* are absorption coefficient, Planck constant, light frequency and reflectance with the reflectance at 1000 nm set at 100%, respectively. As shown in Fig. 5b, the band gap energies of the TiO_2_ species in FK/TiO_2_ and RK/TiO_2_ are 3.21 and 3.18 eV, respectively. For comparison, the bandgap energy of bare TiO_2_ nanoparticles, calculated form the corresponding plot of (*αhv*)^2^ vs. photon energy (*hv*), is 3.2 eV. The results indicate that FK/TiO_2_ possesses larger band gap energy, which can be attributed to the smaller grain size and the more intense interaction between TiO_2_ and FK. It is conducive to the enhancement of photocatalytic performances.

The photoluminescence (PL) spectra of the samples at the excitation wavelength of 254 nm are shown in Fig. 5c. The PL peaks of clays/TiO_2_ are much lower than that of bare TiO_2_, thus suggesting the less recombination of photogenerated electrons and holes, which would lead to improved photocatalytic activity. FK/TiO_2_ nanocomposites have the intimate and uniform interfacial contact, in which photogenerated electrons from TiO_2_ conduction-band are injected rapidly into FK across the particles-nanosheets heterostructure interface. The smaller particles size and more efficient charge separation is achieved and consequently leads to higher photocatalytic activity, compared with RK/TiO_2_ and bare TiO_2_ nanoparticles.

### Photocatalytic properties

**Figure 6:**
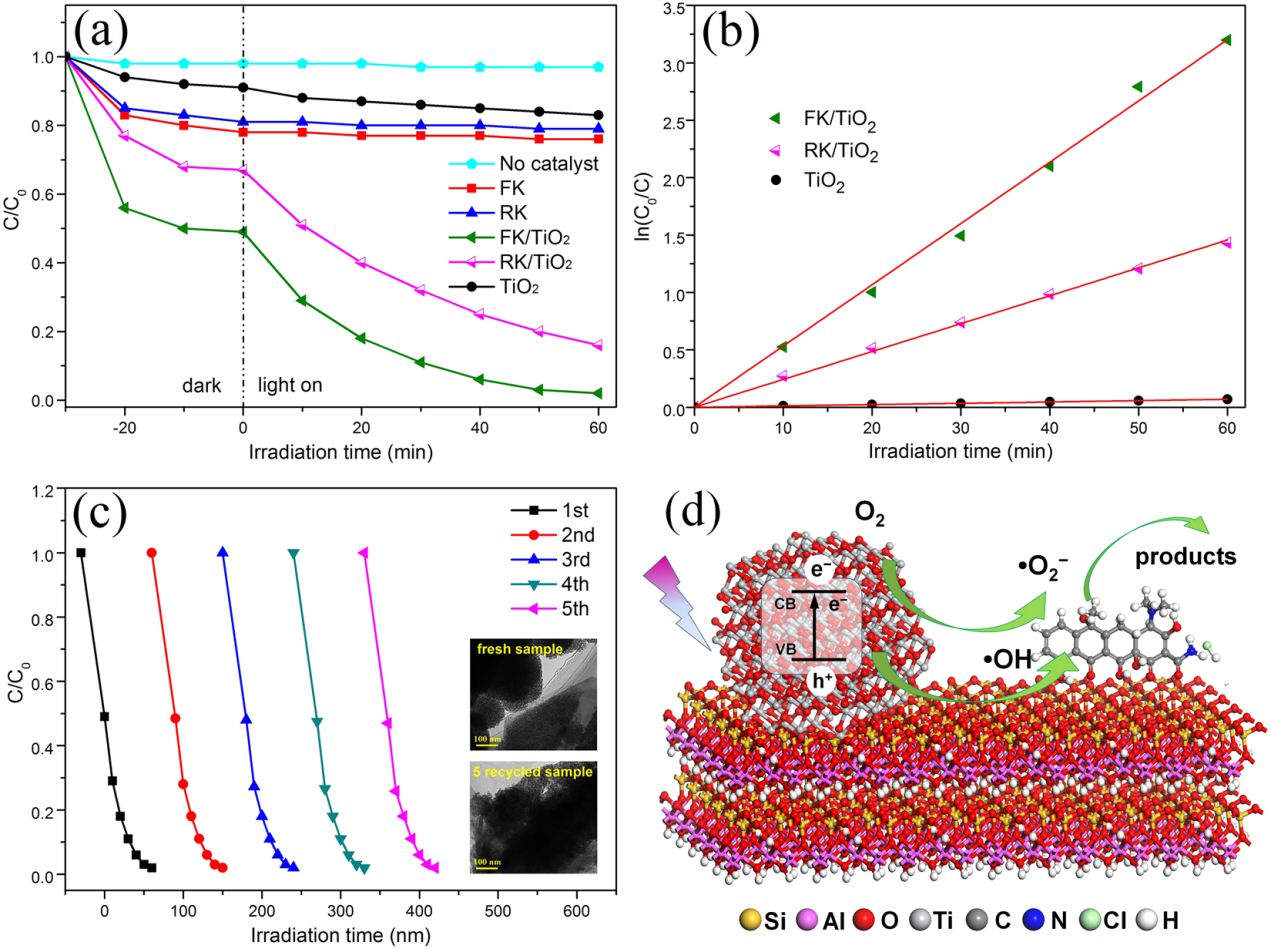
Photocatalytic properties and reaction mechanism of the samples. (**a**) Photocatalytic degradation curves of different samples, (**b**) pseudo-first-order plot with FK/TiO_2_, FK/TiO_2_ and bare TiO_2_ nanoparticles. (**c**) Reusability of FK/TiO_2_ nanocomposite for photocatalytic decomposition of tetracycline hydrochloride (the insets are the TEM images of fresh and recycled samples). (**d**) Schematic illustration of photocatalytic reaction mechanism of clays/TiO_2_ nanocomposites.

The photocatalytic performances of as-prepared photocatalysts were measured to degrade antibiotics under UV light irradiation (Fig. 6). Figure 6a shows the degradation curves of tetracycline hydrochloride using FK, RK, FK/TiO_2_, RK/TiO_2_ and bare TiO_2_ photocatalysts. For the blank experiment analysis, the result shows that tetracycline hydrochloride can be hardly degraded after 60 min under UV light irradiation without catalysts, excluding the possibility of self-photolysis in this system. The bare TiO_2_, FK and RK exhibit 9, 22 and 19% of tetracycline hydrochloride adsorption after 30 min dark adsorption equilibrium, respectively. Upon UV light irradiation, the tetracycline hydrochloride can be slightly degraded with bare TiO_2_, while there is no degradation by FK and RK. The photodegradation activity is enhanced by supporting TiO_2_ nanoparticles on the kaolinites, and FK/TiO_2_ composites show the highest photodegradation activity. After irradiation for 60 min, the photodegradation rate of FK/TiO_2_ composite is 98%, but only 84% for the RK/TiO_2_. It is reported that photocatalytic decomposition of tetracycline hydrochloride follows the pseudo-first-order reaction kinetics^40^. As shown in Fig. 6b, the rate constant for bare TiO_2_ is very small compared to that for clays/TiO_2_ composites, and the rate constant of FK/TiO_2_ is higher than that of RK/TiO_2_. The enhanced photocatalytic activity for FK/TiO_2_ composite can be attributed to the large surface area, decreased particle size and increasing density of active sites.

The stability and recyclability of FK/TiO_2_ nanocomposite were evaluated by monitoring the reactivity of FK/TiO_2_ during five reaction cycles. As shown in Fig. 6c, the photocatalytic activity of FK/TiO_2_ can be easily recovered, and the photodegradation activity has no obvious decrease after five successive cycles. Moreover, the postmortem study shows that there is no significant changes for the supported structure of FK/TiO_2_ after five reaction cycles, which indicates FK/TiO_2_ has good stability. The good stability of FK/TiO_2_ catalyst could be ascribed to the intense interaction between TiO_2_ and FK, which can immobilize the active sites in photocatalysis. The well stability would greatly promote their practical application to eliminate the antibiotics.

## Discussions

Based on the above results, a possible mechanism to explain the enhancement of the photocatalytic properties of the clays/TiO_2_ nanocomposites is depicted in Fig. 6d. The proposed mechanism is the synergetic effects between the kaolinite clays and supported TiO_2_ nanoparticles. The TiO_2_ clusters act as a light absorber, while the kaolinite clays with different morphology are the physical adsorbents of tetracycline hydrochloride molecules. In the dark, tetracycline hydrochloride molecules could be effectively adsorbed around the clays/TiO_2_ nanocomposites and reach adsorption-desorption equilibrium on their surface, which could facilitate the photocatalytic reactions. TiO_2_ nanoparticles supported on the clays could be excited to yield electrons (e^−^) and holes (h^+^) (Eq. (1)). These photo-induced electrons-holes reacted with oxygen molecules (O_2_), H_2_O or hydroxyl groups on the surface of kaolinites to yield hydroxyl radicals (^•^OH) and superoxide radical anions (O_2_^•−^) (Eq. (2–11))^41^. These active species with strong oxidizability directly or indirectly interacted with tetracycline hydrochloride molecules already adsorbed on FK/TiO_2_ and RK/TiO_2_ in aqueous solutions (Eq. (12–13))^37^. The complete equations of mechanism reactions are as follows:

1 $${{\rm{TiO}}}_{2}+{\rm{hv}}\to {{\rm{TiO}}}_{2}({{\rm{h}}}^{+}+{{\rm{e}}}^{-})$$

Reaction involving valence band h^+^

2 $${{\rm{TiO}}}_{2}({{\rm{h}}}^{+})+{{\rm{H}}}_{2}{\rm{O}}\to {{\rm{TiO}}}_{2}{+}^{\bullet }\,{\rm{OH}}+{{\rm{H}}}^{+}$$

3 $${{\rm{TiO}}}_{2}({{\rm{h}}}^{+})+{{\rm{OH}}}^{-}\to {{\rm{TiO}}}_{2}{+}^{\bullet }\,{\rm{OH}}$$

4 $${{\rm{TiO}}}_{2}({{\rm{h}}}^{+})+2{{\rm{H}}}_{2}{\rm{O}}\to {{\rm{TiO}}}_{2}+{{\rm{H}}}_{2}{{\rm{O}}}_{2}+2{{\rm{H}}}^{+}$$

Reaction involving conduction band e^−^

5 $${{\rm{TiO}}}_{2}({{\rm{e}}}^{-})+{{\rm{O}}}_{2}\to {{\rm{TiO}}}_{2}+{{{\rm{O}}}_{2}}^{\bullet -}$$

6 $${{{\rm{O}}}_{2}}^{\bullet -}+{{\rm{H}}}^{+}\to {{{\rm{HO}}}_{2}}^{\bullet }$$

7 $${{\rm{TiO}}}_{2}({{\rm{e}}}^{-})+{{{\rm{HO}}}_{2}}^{\bullet }\to {{\rm{TiO}}}_{2}+{{{\rm{HO}}}_{2}}^{-}$$

8 $${{\rm{TiO}}}_{2}({{\rm{e}}}^{-})+{{{\rm{O}}}_{2}}^{\bullet }+2{{\rm{H}}}^{+}\to {{\rm{TiO}}}_{2}+{{\rm{H}}}_{2}{{\rm{O}}}_{2}$$

9 $${{\rm{TiO}}}_{2}({{\rm{e}}}^{-})+{{\rm{H}}}_{2}{{\rm{O}}}_{2}\to {{\rm{TiO}}}_{2}{+}^{\bullet }\,{\rm{OH}}+{{\rm{OH}}}^{-}$$

10 $${{{\rm{O}}}_{2}}^{\bullet -}+{{\rm{H}}}_{2}{{\rm{O}}}_{2}{\to }^{\bullet }\,{\rm{OH}}+{{\rm{OH}}}^{-}+{{\rm{O}}}_{2}$$

11 $$2{{{\rm{HO}}}_{2}}^{\bullet }\to {{\rm{O}}}_{2}+{{\rm{H}}}_{2}{{\rm{O}}}_{2}$$

12 $$R \mbox{-} H({\rm{TC}}\,{\rm{molecules}}){+}^{\bullet }\,{\rm{OH}}\to {{\rm{R}}}^{\bullet }+{{\rm{H}}}_{2}{\rm{O}}$$

13 $$R \mbox{-} H({\rm{TC}}\,{\rm{molecules}})+{{\rm{h}}}^{+}\to {{\rm{R}}}^{+\bullet }\to {\rm{Degradation}}\,{\rm{products}}$$

Above-mentioned photocatalytic mechanism for tetracycline hydrochloride photodegradation, the surface adsorption and light-induced charge transfer are two primary factors affecting the photocatalytic activity. Strong adsorptivity contributes to the concentration of antibiotics from the solvent and consequently improves the photocatalytic activity. By comparison with FK/TiO_2_ and RK/TiO_2_ nanocomposites, FK/TiO_2_ exhibits much stronger surface adsorption, compared to that of RK/TiO_2_, which can originate from the large specific surface area. Moreover, the TiO_2_ nanoparticles exhibit much more uniform and smaller for FK/TiO_2_, while aggregation seriously for RK/TiO_2_, and FK/TiO_2_ has relatively higher loading efficiencyof TiO_2_ nanoparticles than that of RK/TiO_2_, which can result in higher light-induced charge transfer, and finally enhance the photocatalytic activity. It is clear that the efficient photodegradation performance of FK/TiO_2_ can be attributed to the synergistic effect of better adsorption capability and more active photocatalysis TiO_2_ species. The specific surface area and the grain size of TiO_2_ nanoparticles are strongly depended on the morphology of the kaolinite supports. Therefore, it is important to develop advanced technologies to control the particles morphology so that to enhance the catalytic performances.

In summary, this paper proposed a facile sol-gel method to synthesize clays/TiO_2_ nanocomposites with high catalytic activity and stability. Based on the natural different morphologies and unique layered structure, the efficient assembly and high-density dispersion of uniform TiO_2_ nanoparticles were successfully achieved on the surface of kaolinite clays. Degradation of antibiotics using clays/TiO_2_ catalysts was investigated to elucidate the effects of kaolinite microstructure, morphology and dimensionality for a significant suppression of the TiO_2_ nanoparticle aggregation during the reaction. FK/TiO_2_ exhibited remarkably enhanced photoactivities toward degradation of tetracycline hydrochloride, and the overall degradation rate was up to 98% after light irradiation for 60 min. It could be attributed to the two-dimensional morphology, stronger surface adsorptivity, higher loading efficiency, smaller grain size and intimate interfacial contact. Therefore, our insight into the comparison of different dimensionality of kaolinite clays to photocatalytic performance could be a reference function to the similar investigation, and clays/TiO_2_ composites have great potential applications to eliminate effectively antibiotics.

## Methods

### Materials

The kaolinite used was raw kaolin obtained from Fujian and Guangdong, China. The chemical compositions of flake-like kaolinite (FK) and rod-like kaolinite (RK) were listed in Table 1 and showed that kaolinites with different morphologies possessed the similar chemical composition. Other reagents were purchased from Sinopharm Chemical Reagent Co. Ltd. All reagents were analytical grade and used without further purification.

### Preparation

TiO_2_/kaolinite nanocomposite materials were prepared through a facile precipitation method. In a typical synthesis, 0.2 g kaolinite was added to a mixture constituted by 30.0 mL of ethanol and 0.6 mL of deionized water under dispersing in the ultrasonic bath for 30 min. Subsequently, 3 mL of tetrabutyl titanate (TBOT) dissolved in 5 mL of ethanol were put drop-wise into the kaolinite suspensions. After continuous stirring for 2 h at 80 °C, the precipitates were collected by centrifugation and subsequently washed with deionized water repeatedly. The resultant products were dried overnight at 80 °C. Finally, the samples were calcined under 450 °C for 3 h in air with a heating rate of 5 °C/min. For comparison, the bare TiO_2_ nanoparticles were also obtained via a similar process as TiO_2_/kaolinite composite without adding kaolinite.

### Photocatalytic degradation experiments

The photocatalytic activity of the catalysts for tetracycline hydrochloride was investigated at ambient temperature. In a typical process, 50 mg of catalyst was dispersed in 50 mL of 30 mg/L tetracycline hydrochloride solution. The suspension was vigorously stirred in the dark for 30 min to reach the adsorption-desorption equilibrium, and then irradiated with ultraviolet light. Afterwards, 2.5 mL aliquots of the reaction mixtures were collected and the catalyst was removed from the solution using a 0.45 μm cellulose acetate membrane filter. The tetracycline hydrochloride concentrations in the filtrates were measured at 380 nm using the UV-vis spectrophotometer. The stability of catalyst was evaluated by the catalytic cycle test. At the end of each cycle, the suspension was filtered and the catalyst was tested in the next cycle.

### Characterization

The chemical composition of kaolinite minerals and nanocomposites were determined using X-ray fluorescence (XRF) spectrometer. The structural characteristics of the samples were examined by X-ray diffraction (XRD, Rigaku D/MAX2550VB+) using Cu K*α* radiation (*λ* = 0.15406 nm) at a scanning rate of 0.02 °/s with a voltage of 40 kV and 40 mA. The microstructures of the samples were observed using a scanning electron microscopy (SEM, FEI Quanta-200) with an accelerating voltage of 5 kV, a transmission electron microscopy (TEM, JEOL JEM-2100F) and high-resolution transmission electron microscopy (HRTEM, JEOL JEM-3010) operating at 200 kV. The TiO_2_ clusters were identified by X-ray photoelectron spectroscopy and their size distributions were determined by counting the sizes of TiO_2_ clusters on TEM images taken from different places. The textural properties of the samples were determined by N_2_ porosimetry. The N_2_ adsorption-desorption isotherms were recorded at 77 K and analyzed using an ASAP 2020 Surface Area analyzer (Micromeritics Co. Ltd.). The specific surface areas were calculated using the Brunnauer-Emmett-Teller (BET) equation, and estimates of the pore size distributions were deduced by means of Barrett-Joyner-Halenda (BJH) methods. The zeta potential of the samples at different pH levels was measured on a zeta potential analyzer (Zetasizer Nano ZS90, Malvern) at solids content of about 0.1% in distilled water. The interface characteristics and their chemical nature were studied by Fourier transform infrared (FTIR, Nicolet Nexus 670) and X-ray photoelectron spectroscopy (XPS, ESCALAB 250). Diffuse reflectance ultraviolet-visible (UV-vis) spectra were obtained on a Hitachi (Shimadzu) Model UV-2450 spectrophotometer. The Photoluminescence (PL) spectra were measured on a Hitachi F-4500 fluorescence spectrometer at room temperature using a Xe lamp with a wavelength of 254 nm as the excitation source. For the photocatalytic activity evaluation, a 150 W Xe lamp equipped with an optical cutoff filter (λ < 400 nm) was used as the light source, and the concentration of photodegraded tetracycline hydrochloride solution were recorded by a UV-vis spectroscopy (UV2450).

### Data availability statement

The datasets generated during and/or analyzed during the current study are available from the corresponding author on reasonable request.
